# Performance evaluation of a newly developed high‐resolution, dual‐head animal SPECT system based on the NEMA NU1–2007 standard

**DOI:** 10.1120/jacmp.v15i6.4936

**Published:** 2014-11-08

**Authors:** Vahideh Moji, Navid Zeraatkar, Mohammad Hossein Farahani, Mahmood Reza Aghamiri, Salar Sajedi, Behnoosh Teimourian, Pardis Ghafarian, Saeed Sarkar, Mohammad Reza Ay

**Affiliations:** ^1^ Research Center for Molecular and Cellular Imaging Tehran University of Medical Sciences Tehran; ^2^ Department of Radiation Medicine Engineering Shahid Beheshti University Tehran; ^3^ Parto Negar Persia Co. Tehran; ^4^ Chronic Respiratory Diseases Research Center National Research Institute of Tuberculosis and Lung Diseases (NRITLD), Shahid Beheshti University of Medical Sciences Tehran; ^5^ PET/CT and Cyclotron Center Masih Daneshvari Hospital, Shahid Beheshti University of Medical Sciences Tehran; ^6^ Research Center for Science and Technology in Medicine Tehran University of Medical Sciences Tehran; ^7^ Department of Medical Physics and Biomedical Engineering Tehran University of Medical Sciences Tehran Iran

**Keywords:** small‐animal SPECT, performance evaluation, HiReSPECT, parallel‐hole, resolution recovery

## Abstract

Small‐animal single‐photon emission computed tomography (SPECT) system plays an important role in the field of drug development and investigation of potential drugs in the preclinical phase. The small‐animal High‐Resolution SPECT (HiReSPECT) scanner has been recently designed and developed based on compact and high‐resolution detectors. The detectors are based on a high‐resolution parallel hole collimator, a cesium iodide (sodium‐activated) pixelated crystal array and two H8500 position‐sensitive photomultiplier tubes. In this system, a full set of data corrections such as energy, linearity, and uniformity, together with resolution recovery option in reconstruction algorithms, are available. In this study, we assessed the performance of the system based on NEMA‐NU1–2007 standards for pixelated detector cameras. Characterization of the HiReSPECT was performed by measurement of the physical parameters including planar and tomographic performance. The planar performance of the system was characterized with flood‐field phantom for energy resolution and uniformity. Spatial resolution and sensitivity were evaluated as functions of distance with capillary tube and cylindrical source, respectively. Tomographic spatial resolution was characterized as a function of radius of rotation (ROR). A dedicated hot rod phantom and image quality phantom was used for the evaluation of overall tomographic quality of the HiReSPECT. The results showed that the planar spatial resolution was ~1.6mm and ~2.3mm in terms of full‐width at half‐maximum (FWHM) along short‐ and long‐axis dimensions, respectively, when the source was placed on the detector surface. The integral uniformity of the system after uniformity correction was 1.7% and 1.2% in useful field of view (UFOV) and central field of view (CFOV), respectively. System sensitivity on the collimator surface was 1.31cps/μCi and didn't vary significantly with distance. Mean tomographic spatial resolution was measured ∼1.7 mm FWHM at the radius of rotation of 25 mm with dual‐head configuration.

The measured performance demonstrated that the HiReSPECT scanner has acceptable image quality and, hence, is well suited for preclinical molecular imaging research.

PACS number: 87.57.U

## I. INTRODUCTION

Animal models of human diseases are widely used in the field of drug development and investigating potential therapies and gene research in the preclinical phase.^(1)^ Among various laboratory animals used in research studies, small animals such as mice and rats have advantages over large animals due to low cost, rapid breeding cycles, and high genetic homology with humans.^2^, ^3^ Noninvasive *in vivo* imaging of animals using different techniques, such as positron emission tomography (PET) and single‐photon emission computed tomography (SPECT), enables the acquisition of a complete set of data from every animal. Therefore, such techniques reduce the number of laboratory animals used, along with the reduction of variability in the subject of the study.^(4)^ The advantages of SPECT compared with PET are greater radiotracer availability, lower cost, and longer‐lived isotopes.^(5)^

Over the recent years, animal SPECT systems have been developed in order to achieve high‐resolution and high‐sensitivity systems. Several small‐animal SPECT systems with compact detectors have been developed in different groups and are available commercially. Loudos et al.^(6)^ developed a compact gamma camera consisting of a pixelated cesium iodide (sodium‐activated) (CsI(Na)) crystal array coupled to H8500 position‐sensitive photomultiplier tubes (PSPMTs) and a parallel‐hole collimator for dynamic studies in small animals. They achieved spatial resolution of 1.6 mm full‐width at half‐maximum (FWHM) and 58.5 cps/MBq sensitivity. Xi et al.^(7)^ constructed portable dual‐gamma camera system based on H8500 square PSPMTs coupled to sodium iodide (thallium‐activated) (NaI(Tl)) crystal arrays. Preliminary SPECT phantom imaging was performed with a single detector equipped with a high‐resolution parallel hole collimator having 0.2mm×0.2mm square openings and 0.05 mm septa thickness. The system has spatial resolution of 2.2 mm FWHM at 1 cm, sensitivity of 149 cps/MBq, energy resolution of 10.8%, and spatial uniformity less than 3%. Magota et al.^(8)^ developed Inveon preclinical small‐animal PET/SPECT/CT scanner which contains pixelated NaI(Tl) crystal array with dimension of $2.0\times 2.0\times 10\;{\text{mm}}^{3}$, PSPMT readout, and 0.5 to 3 mm single pinhole collimators. Tomographic spatial resolution and sensitivity of the system for 0.5 mm pinhole collimator was reported 0.84 mm and 35.3 cps/MBq, respectively. A‐SPECT^9^, ^10^ was the first commercial small‐animal SPECT scanner available. The system had interchangeable single pinhole apertures and vertically oriented animal rotary stage. The second generation system, X‐SPECT, was similar to A‐SPECT, but having rotating detectors and horizontally positioned stationary animal bed in addition to an integrated CT. Following the widespread use and commercial availability of small‐animal SPECT scanners, the National Electrical Manufacturers Association (NEMA) published its NU1–2007 standards providing consistent and standardized methodology for measuring scanner performance parameters for pixelated systems.^(11)^ The aim of this study was to assess the performance of a pixelated high‐resolution animal SPECT in planar and tomographic modes, based on the NEMA standard.

## II. MATERIALS AND METHODS

### A. System description

**Figure 1:**
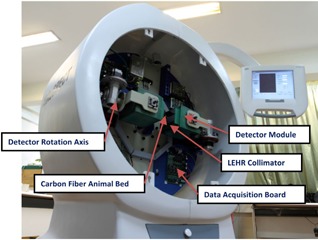
The developed dual‐head, high‐resolution animal SPECT system called HiReSPECT.

The newly developed HiReSPECT scanner (Fig. 1) consists of two planar heads based on a pair of H8500C PSPMT (Hamamatsu Photonics Co., Hamamatsu City, Japan) with $49\times 49\;{\text{mm}}^{2}$ active areas for each head. H8500C is a 12‐stage tube with a gain of 10^6^ and an 8×8 array of anode pads. A 46×89 array of CsI(Na) scintillator crystal (Hilger Crystals, Margate, UK) with the pixel dimensions of 1mm×1mm×5mm (1.2 mm pitch) was utilized. Low‐energy, high‐resolution lead parallel‐hole collimators with 1.2 mm hexagonal holes, 34 mm thickness, and 0.2 mm septal thickness (Nuclear Fields Co., St. Mary's, Australia) have been used.^(12)^

A full set of calibrations was performed in the system. First, using a dedicated bar phantom, a look‐up table (LUT) was generated for positioning. Then, flood image is obtained to perform energy and uniformity corrections. The correction and calibration methods can be found in more detail in Sajedi et al.^(12)^ For SPECT studies, the projection images of each view are stored in a 38×80 matrix. Center of rotation (COR) alignment is carried out to the projection matrix before image reconstruction. The procedure involves imaging of a line source at the axis of rotation. Then COR error and the axial deviation of the image for each detector are calculated with the analysis method proposed by the NEMA standard. Then, these values are used for correction of each projection matrix.

A dedicated code was developed to perform image reconstruction, including resolution recovery in the system, employing a modified maximum‐likelihood expectation‐maximization (MLEM) algorithm with pixel‐driven, rotation‐based, resolution‐recovery technique. Matrix size of the reconstructed image is 128×128×240 resulting in voxel size of $0.4\times 0.4\times 0.4\;{\text{mm}}^{3}$. The resolution recovery improves spatial resolution of the system by modeling the collimator‐detector response function (CDRF) of the system during image reconstruction. CDRF was characterized via a pixel‐driven method, involving capillary sources at varying distances from the head within the field of view (FOV) and subsequent fitting by independent Gaussian functions.^(12)^

### B. NEMA performance measurement

#### B.1 System spatial resolution

The system spatial resolution was measured using some capillary tubes (1.1 mm inner diameter and 5 cm length) filled with Tc‐99m solution which was placed in the useful field of view (UFOV). The line source was placed at different distances from the collimator from 0 mm to 120 mm with 10 mm increments. Spatial resolution was calculated as FWHM of the line spread function (LSF). FWHMs were determined in the horizontal and vertical directions of the image, as well as average and standard deviation of FWHMs in different positions of the image.

To calculate the FWHM at a given distance, the LSF was constructed from the line profile of the capillary tube. In the next step, the LSF was fitted using the least squares minimization to a Gaussian model (Eq. (1)). The Gaussian fit was used to estimate the FWHM of the LSF (Eq. (2)).

(1) $$f(x)=a_{1}\times \text{exp}\left[-\frac{{\left(x-b_{1}\right)}^{2}}{2\sigma^{2}}\right]$$

(2) FWHM≈2.35σ

#### B.2 System uniformity

The system uniformity was measured with a flood‐field phantom with dimensions of $110\times 60\times 5\;{\text{mm}}^{3}$ filled with Tc‐99m solution. The phantom was sufficiently large to cover the detector FOV and was placed in direct contact with the collimator. The uniformity of the system was evaluated with and without uniformity correction. The flood‐field image, after removing the marginal pixels, was convolved with the smoothing filter recommended by NEMA.^(11)^ The integral uniformity is defined as the difference between the maximum and minimum pixel values within central field of view (CFOV) and UFOV of the detector. Differential uniformity is specified as the largest difference between two pixels within any set of three contiguous pixels in a row or column of the flood‐field image. Uniformity is calculated as follows:^(11)^

(3) $$\text{Uniformity}(\%)=\frac{\text{Maximum}-\text{Minimum}}{\text{Maximum}+\text{Minimum}}\times 100$$

where *Maximum* is the maximum count *and Minimum* is the minimum count found in any pixel within the specified area.

#### B.3 Energy resolution

The energy resolution was measured while the collimator was mounted and the detector illuminated with the same flood‐field source used for uniformity characterization. Energy resolution was determined after energy calibration. For each crystal, an energy spectrum was acquired during the acquisition; an automated algorithm found the photopeak channel in each spectrum and recorded it in an energy LUT A Gaussian fitting was applied to the data of the energy spectra of each crystal array. FWHM of energy peak for each crystal element was calculated and divided by the energy value which was 140 keV. The energy resolution was assessed by the average of all FWHMs of the energy peaks.

#### B.4 System sensitivity

The system sensitivity was measured as a function of distance, ranging from 0 cm to 10 cm. A cylindrical phantom with an inner diameter of 32 mm and 5 mm thickness was filled with 2 mci (74 MBq) Tc‐99m. Then, the phantom was placed at 0 cm, 5 cm, and 10 cm distant from the collimator surface and count rate was measured for a period of 300 s. Counts were decay‐corrected for the Tc‐99m activity using Eq. (4).^(11)^ The system sensitivity was calculated in terms of cps/μCi using Eq. (5).^(11)^

(4) $$R_{i}=C_{i}\text{exp}\left(\frac{\left(T_{i}-T_{\text{cal}}\right)}{T_{\text{half}}}\text{ln}2\right)\times \left(\frac{\text{ln}2}{T_{\text{half}}}\right){\left(1-\text{exp}\left(-\frac{T_{\text{acq},i}}{T_{\text{half}}}\text{ln}2\right)\right)}^{-1}$$

(5) $$\text{Sensitivity}=\frac{R_{i}}{A_{\text{cal}}}$$

where $R_{i}$ is decay‐corrected count rate, $C_{i}$ is summed counts in the ith image, $T_{i}$ is start time of the ith acquisition, $T_{\text{acq},i}$ is duration of the ith acquisition, $T_{\text{half}}$ is half‐life of Tc‐99m (21672 s), and $A_{\text{cal}}$ is the amount of radioactivity in the phantom at time of $T_{\text{cal}}$.

#### B.5 Tomographic spatial resolution

Three Tc‐99m point sources (inner diameter: 1.1 mm) were placed in the FOV; one at the center of FOV and two others placed 10 mm away from the axis of rotation. Data acquisition was performed through 60 views over 360° for each head in the single‐head mode and over 180°/head in the dual‐head mode. Acquisition time for the first view was adjusted to 60 s. The capability of decay compensation was included in our acquisition protocol by accordingly increasing the acquisition times for each subsequent view so that the latest view took 69 s. Data were reconstructed using filtered back‐projection technique, with and without resolution recovery. A Gaussian function was fitted to central, radial, and tangential count profiles of each of the reconstructed point sources. FWHMs in different radii of rotation (ROR) 25 mm, 35 mm, 55 mm, 75 mm, and 85 mm were calculated.

### C. Phantoms

#### C.1 Dedicated hot rod phantom

**Figure 2:**
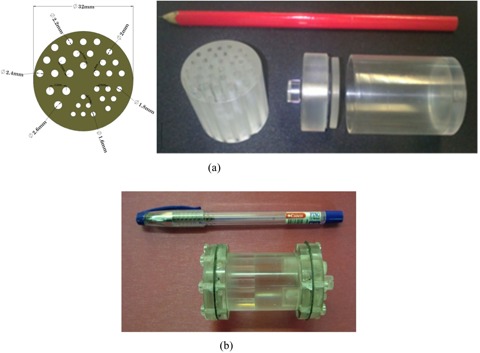
The dedicated in‐house hot rod phantom (a) designed for tomographic spatial resolution characterization; (b) the image quality phantom recommended by NEMA NU‐4 2008.

A dedicated phantom was designed and fabricated from Plexiglass to assess the reconstructed image quality of the system. As shown in Fig. 2(a), the phantom had an outside diameter of 32 mm and 35 mm height, and consisted of six sections of hot rods with diameters 1.6, 1.8, 2, 2.2, 2.4, and 2.6 mm. The phantom was filled with 2 mCi (74 MBq) of Tc‐99m.

#### C.2 Image quality phantom

Image‐quality characteristics were assessed using NEMA NU 4–2008 image‐quality (IQ) phantom.^(13)^ The phantom (Fig. 2(b)) was made from Plexiglass with internal dimensions of 50 mm in length and 30 mm in diameter. The phantom consists of three parts: the main phantom body composed of a fillable cylindrical chamber with 30 mm diameter and 30 mm length, and a solid part 20 mm in length with five fillable rods has been drilled through (at 7 mm from the center) with diameters of 1, 2, 3, 4, and 5 mm, a uniform region, and a lid, that attaches to the large uniform region end of the phantom, supports two cold region chambers. One of these chambers filled with nonradioactive water to simulate attenuation only, and the other filled with air. These chambers are composed of hollow cylinders 15 mm in length, 8 mm inner diameter, and 1 mm wall thickness (Fig. 2(b)).

For data acquisition, the phantom was filled uniformly with 5 mCi (185 MBq) Tc‐99m dissolved in normal saline. The data analysis was performed to obtain values on image uniformity, recovery coefficients, and accuracy of data corrections. 1) Uniformity of the reconstructed image was evaluated as a percentage standard deviation of cylindrical volume of interest (VOI) (75% of the physical diameter, 10 mm height) over the center of the uniform region of the image quality phantom. The maximum, minimum, and mean values in this VOI were measured, too. 2) Activity recovery coefficients (RC) for hot rods were measured as the ratio of the maximum values in each plane of the VOIs (twice the physical diameter of the rods, 10 mm height) over the calculated mean phantom concentration in the uniformity test. 3) Spill‐over ratios (SORs) were calculated as the ratio of the mean counts in each cold region VOI (diameter 4 mm, 7.5 mm in length) to the mean of the hot uniform area. The standard deviation of the RCs and SORs was calculated based on Eq. 6:

(6) $${\text{SD}}_{\text{RC}}=\sqrt{{\left(\frac{{\text{SD}}_{\text{lineprofile}}}{{\text{average}}_{\text{lineprofile}}}\right)}^{2}+{\left(\frac{{\text{SD}}_{\text{background}}}{{\text{average}}_{\text{background}}}\right)}^{2}}.$$

## III. RESULTS

### A. System spatial resolution

**Figure 3:**
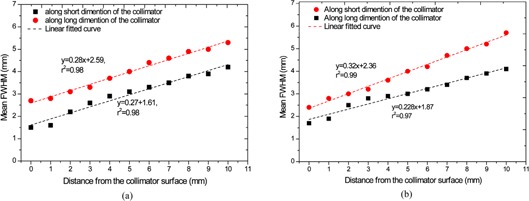
FWHM as a function of distance from the collimator surface in vertical and horizontal directions: (a) detector 1; (b) detector 2.

The average resolution on the collimator surface was ∼1.6mm and ∼2.3mm along the short and long dimensions, respectively. Figure 3 depicts the measured extrinsic resolution (in terms of FWHM) as functions of distance from the collimator surface. As expected for parallel‐hole collimation, the spatial resolution has a linear trend with distance.^(14)^

### B. System uniformity

**Figure 4:**
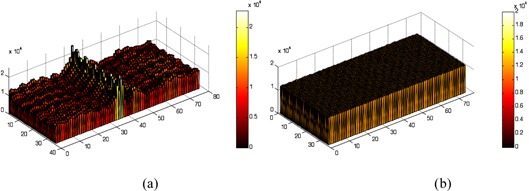
The two‐dimensional profiles of the flood images without (a) and with (b) uniformity correction, respectively.

Figure 4 shows the two‐dimensional profiles of the images before and after uniformity correction for one of the detectors (detector 1). The uniformities of the system were summarized in Table 1. The results illustrate the improvement of image uniformity after correction in the system.

**Table 1:** Uniformity analysis before and after uniformity correction.

*Detector 2*				*Detector 1*				
*With Uniformity Correction*		*Without Uniformity Correction*		*With Uniformity Correction*		*Without Uniformity Correction*		
*CFOV*	*UFOV*	*CFOV*	*UFOV*	*CFOV*	*UFOV*	*CFOV*	*UFOV*	
1.3	1.4	50.2	50.2	1.51	1.74	54	55.4	Integral Uniformity (%)
0.83	0.93	34.1	31.6	1.26	1.31	42.1	29.6	Differential Uniformity in the Columns (%)
0.93	0.95	8.9	8.2	1.26	1.28	11.6	9.1	Differential Uniformity in the Rows (%)

### C. Energy resolution

The total energy spectrum is affected by the nonuniform response of the PMTs, as well as the loss of signal in the area between them. However, by calculating the energy spectrum of each crystal cell and by normalizing them using the peak shift method, an improved total normalized energy spectrum was obtained. The average intrinsic energy resolution of 19.7% and 18.6% was measured for detector 1 and detector 2, respectively, at Tc‐99m photon energy.

### D. System planar sensitivity

System sensitivity of 1.32cps/μCi and 1.31cps/μCi on the collimator surface was measured with and without uniformity correction for detector 1, respectively; for detector 2, the system sensitivity was 1.25cps/μCi and 1.21cps/μCi, respectively, with and without uniformity correction. Planar sensitivities of the system in distances of 4, 8, and 12 cm are 1.22, 1.2, and 1.1cps/μCi for detector 1 and 1.18, 1.16, and 1.15cps/μCi for detector 2, respectively. As expected for parallel‐hole collimation, a small decrease is observed when distance is increased.

### E. Tomographic spatial resolution

**Figure 5:**
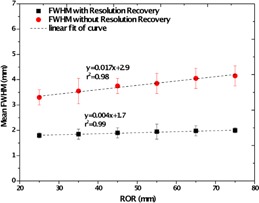
Mean tomographic spatial resolution (mm) as a function of ROR, with and without resolution recovery.

The tangential, radial, and central spatial resolution of the system with and without resolution recovery is presented in Table 2. Figure 5 shows the average of FWHM values and standard deviations of the FWHMs in the dual‐head mode with and without resolution recovery as a function of ROR (mm).

**Table 2:** Tangential, radial, and central spatial resolution (FWHM in mm) of the system at ROR of 25 mm with and without resolution recovery.

*With Resolution Recovery*			*Without Resolution Recovery*			
*Dual Head*	*Head 2*	*Head 1*	*Dual Head*	*Head 2*	*Head 1*	
1.7	1.7	1.8	3.2	3.0	3.2	Tangential (mm)
1.7	1.8	1.7	3.2	3.5	2.9	Radial (mm)
1.8	1.8	1.8	3.3	3.3	3.2	Central (mm)

### F. Dedicated hot rod phantom

**Figure 6:**
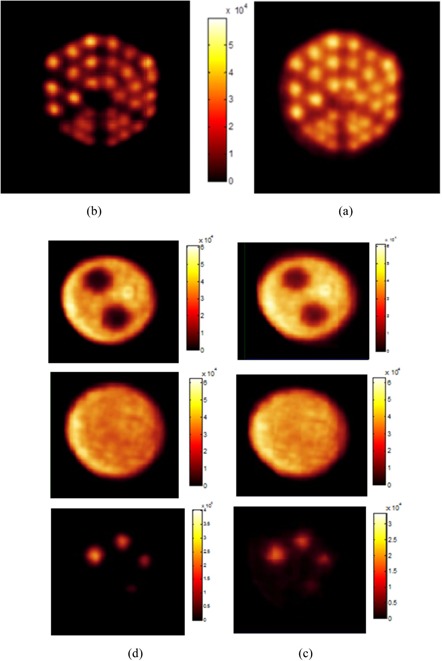
Transaxial images of a dedicated hot‐rods phantom without resolution recovery (a) and with resolution recovery (b), respectively. Images of NEMA image quality phantom without resolution recovery (c) and with resolution recovery (d), respectively.

Figures 6(a) and 6(b) show a transaxial image of the dedicated hot rod phantom with and without resolution recovery. In these images, the rods in the five first sections (diameters 2.6, 2.4, 2.2, 2.0, and 1.8 mm) are clearly visualized both with and without resolution recovery. The group of rods with a diameter of 1.6 mm cannot be clearly distinguishable without using resolution recovery, while they are distinguishable with resolution recovery.

### G. Image quality phantom

**Figure 7:**
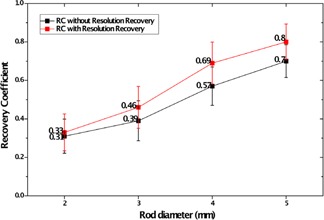
Recovery coefficients and standard deviations for four rods of different sizes ranging from 2 to 5 mm.

Figures 6(c) and 6(d) show the reconstructed images of the NEMA image‐quality phantom with and without resolution recovery including a transaxial view of the five rods, a transverse plane of the nonradioactive chambers, and a transaxial view of the uniform region of the phantom. The mean, maximum, and minimum values in the VOI of uniform region are 39174, 54801, and 28777, respectively. The percentage standard deviation was measured about 8.1% without resolution recovery and 8.5% with resolution recovery. The measured SOR was 0.24 and 0.23 for the water‐ and air‐filled inserts of the image quality phantom, respectively. The RCs and absolute standard deviation for the different rods are illustrated in Fig. 7.

## IV. DISCUSSION & CONCLUSIONS

HiReSPECT, a high‐resolution small‐animal SPECT, was designed, assembled, and characterized for high‐resolution imaging of small animals such as mice and rats in the laboratory. The aim of this study was the performance evaluation of a newly developed dual‐detector small‐animal SPECT (HiReSPECT) imaging system. The evaluation was based on the NEMA NU1–2007 protocol released for investigating performance of systems with pixelated crystal. ^(11)^ In order to evaluate the performance of the correction methods in this system, sensitivity, linearity, and uniformity were assessed before and after the corrections. The results show that the correction algorithms considerably improve the system performance.

The spatial resolution of a pixelated system when using capillary sources for quantification on the low distance from the surface of the collimator highly depends on the relative position of the capillary source and the detector elements. When the capillary source is placed exactly above the center of a physical pixel, the emission signal is shared between fewer pixels, leading to a narrower FWHM. On the other hand, when the source is located between the pixels, the emission signal is shared between a larger numbers of pixels, resulting in a wider FWHM. Also, due to the shape and arrangement of holes in parallel hole collimators, extrinsic spatial resolution depends on the direction of the source on the collimator surface.

It is well known that the energy resolution of pixelated detectors is a function of both pixel size and intrinsic efficiency of the crystal material. Normally, the limited energy resolution in pixelated crystals is due to the fact that there is an amount of light loss between detector pixel elements. So, this configuration of small gamma cameras often has a FWHM energy resolution worse than single crystal scintillation cameras.^15^, ^16^, ^17^, ^18^

The uniformity of the HiReSPECT system with uniformity correction was less than 2%. However, uniformity correction algorithm significantly improved the uniformity of image in the system. It has been concluded that the uniformity of image in the HiReSPECT system is good with comparison to recently developed animal cameras.^7^, ^19^, ^20^, ^21^

The results of the tomographic resolution measured on HiReSPECT system shows that tomographic resolution for dual‐head mode in 25 mm ROR is ∼3.2mm in the central region. The tomographic spatial resolution of 1.7 mm was measured when using resolution recovery‐embedded image reconstruction. Tomographic resolution improves significantly with resolution recovery and does not vary considerably with increasing the ROR.

Figures 6(a) and 6(b) show that the group of rods with a diameter of 1.6 mm could not be resolved without a resolution recovery; this group is fully resolvable when using resolution recovery.

In this study, we used image quality test recommended by NEMA NU4–2008 for image quality characterization of a small animal PET. The %STD which expresses the uniformity of the IQ phantom image is 8.1% without resolution recovery and 8.5% with resolution recovery. Because resolution recovery at low frequencies increases the image contrast, using resolution recovery in reconstruction procedure reduces the uniformity of the system. The recovery coefficients for four different rods varied from 0.3 to 0.7 (Fig. 7) without resolution recovery and 0.3 to 0.8 with resolution recovery. SOR values of water‐ and air‐filled chambers are 0.24 and 0.23 without resolution recovery, respectively, and both of them are 0.17 with resolution recovery. Since the images were not corrected for attenuation and scatter, such high values for SOR were expected. The images of IQ phantom were demonstrated that resolution recovery decreases noise in low frequencies. Hence, cold regions became clearer. Also, the hot rods in these images were fully resolvable when using resolution recovery.

The results demonstrate that the system in its current dual‐head configuration is suitable for dedicated small‐animal SPECT imaging. This system has comparable planar (∼1.9mm) and tomographic (∼1.7mm) spatial resolution in comparison with the other available small‐animal SPECT systems. As was discussed, planar resolution of the HiReSPECT system in near‐field imaging depends on the source position. The methods which can be used to overcome this problem are displacing the capillary tube laterally in 1 mm steps over a distance of 10 mm or two detector elements, or tilting the capillary tube on the detector surface.^22^, ^23^ The achieved uniformity values are less than values of clinical gamma cameras, which usually are <5% uniformity for acceptable human imaging. The sensitivity of the system was assessed ∼1.32cps/μCi. The sensitivity of system strongly depends on collimator designs, energy window, energy resolution, and intrinsic efficiency of crystal, so cannot be compared directly with other systems. However, comparable sensitivity data are available for other gamma camera detector systems.^6^, ^22^, ^24^

Overall, the results of performance measurement in this study show that the HiReSPECT is able to generate high‐quality images for molecular imaging of small animals. The correction techniques used in this system work well and improve the performance of the system. Also, some developments in this system can further improve the overall quality of images. Tomographic and planar spatial resolution can be improved by using pinhole collimator, and sensitivity can be enhanced using multipinhole collimator. Tomographic image uniformity and overall quality of images can be improved by using attenuation and scatter correction techniques. Therefore, the performance capabilities of the system can be improved further using multipinhole collimators, 3D iterative reconstruction algorithms, and the implementation of CT‐based SPECT for quantitative correction procedures.

## ACKNOWLEDGMENTS

This work was supported under grant number 92–01–30–21342, Tehran University of Medical Sciences, Tehran, Iran.
